# Preliminary Study on Piezoelectric Sensor Signals Embedded in Polymeric Samples

**DOI:** 10.3390/s26082412

**Published:** 2026-04-15

**Authors:** Vítor Miguel Santos, Sara Valvez, Beatriz Branquinho Gomes, Maria Augusta Neto, Ana Martins Amaro

**Affiliations:** 1 University of Coimbra, Centre of Mechanical Engineering, Materials and Processes (CEMMPRE-ARISE), Department of Mechanical Engineering, 3040-248 Coimbra, Portugal; sara.valvez@dem.uc.pt (S.V.); beatrizgomes@fcdef.uc.pt (B.B.G.); augusta.neto@dem.uc.pt (M.A.N.); ana.amaro@dem.uc.pt (A.M.A.); 2 University of Coimbra, Interdisciplinary Center for the Study of Human Performance (CIPER), Faculty of Sports Sciences and Physical Education (FCDEF), 3040-256 Coimbra, Portugal

**Keywords:** piezoelectric sensors, additive manufacturing, compression test, wearable technology

## Abstract

Piezoelectric sensors are widely used for force and vibration monitoring in both rigid and flexible structures, yet their performance can be significantly affected by how they are integrated into the host material. Challenges such as limited sensitivity, inconsistent signal transmission, and delays in response remain particularly relevant in flexible components produced by additive manufacturing. Addressing these limitations requires a better understanding of how integration strategies influence sensor behavior. This study presents preliminary experimental results on the performance of commercial piezoelectric ceramic (PZT) sensors embedded in flexible structures fabricated by additive manufacturing (3D printing). Although the current investigation did not assess variability from mass production, repeated testing of each specimen was performed to reduce this potential error. Filaflex Foamy 95A polyurethane (TPU) samples were produced using Fused Filament Fabrication (FFF) technology in two configurations: with and without a cavity for sensor fitting. A minimum of seven valid compression tests, at each condition, were performed, with ten loading and unloading cycles up to 1000 N of force, using an indentation rate of 0.5 mm/s. In most tests, the two configurations showed different peak amplitudes throughout the cycles. Samples with the sensor embedded in the cavity consistently reached peak signal amplitudes more rapidly. In contrast, samples with the sensor positioned on the material surface without a fitting exhibited similar results across all tests and demonstrated a broader signal distribution over time. These findings indicate that the sensor-integration strategy is the primary factor influencing dynamic force transfer, impact sensitivity, piezoelectric response time, and maximum signal magnitude.

## 1. Introduction

The integration of sensors into flexible structures is essential for the development of wearable equipment, systems, and technologies designed for biomechanical monitoring [1,2,3]. Additive manufacturing, or 3D printing, has an important advantage in enabling customized, reproducible biomedical parts and plays an essential role in wearable/healthcare monitoring [4].

In these applications, the performance of the sensor depends not only on its properties but also on the mechanical interaction between the sensor and its support structure. In deformable polymer materials, the chosen integration strategy can substantially affect the charge transfer mechanisms and, as a result, the measured signal [5,6].

Considering all types of sensors, piezoelectric sensors are chosen and often used in dynamic measurements due to their high sensitivity to apply forces and strain variations [7]. When these sensors are embedded in elastomer-behaving materials, such as polyurethane (TPU) produced by additive manufacturing technologies such as Fused Filament Fabrication (FFF), their electrical response can be influenced by mechanical phenomena, such as damping effects, stress redistribution, and strain site heterogeneity. The inclusion of a fitting for the placement of the sensor is a structural variable that can modify the interaction between sensor and material [5,8,9]. The integration also affects the survivability and usable operation range [10]. The fabrication process of the insole is also important, and the definition of the parameters are crucial to link and intertwine with the piezoelectric sensor purpose [11,12].

PZT (Lead Zirconate Titanate) responds well to mechanical loading but can be limited by brittleness, while PVDF (PolyVinylidene Fluoride) extends its working domain under strain [3,10]. On the other hand, the approaches differ in fabrication cost, scalability, and mechanical flexibility. These differences come from distinct material systems and processing routes. They should be carefully considered when assessing the practical applicability and technological potential of each solution [13,14]. And surface-bonded sensors can be fragile and lose serviceability; that is why it is also important to embed a protective sensor [15,16] or also define a cavity to integrate the sensor and make it more durable and protected without losing its advantages.

In order to understand whether or not there is an influence of the sensor position on the sample to be tested, it is necessary to carry out preliminary experimental tests that compare various sensor-integration strategies to allow the evaluation and understanding of the mechanical factors that influence the signal variability and to help in the development of reliable equipment and wearable system designs.

The main objectives of this study were to evaluate: (1) the relationship between the integration of piezoelectric sensors and polymeric materials, more specifically with samples printed in TPU, and (2) the signal difference (or voltage) captured in prototypes with a cavity and without a cavity for sensor fitting. In addition to establishing these objectives, this work provides a comparative assessment of two sensor-integration strategies, demonstrating that the presence of a dedicated cavity leads to faster response times, higher peak voltage amplitudes, and improved signal stability, whereas surface-mounted sensors exhibit broader but less responsive signals. These insights contribute to a better understanding of how structural integration influences dynamic load transfer and signal reliability in flexible additively manufactured systems.

## 2. Materials and Methods

**Figure 1:**
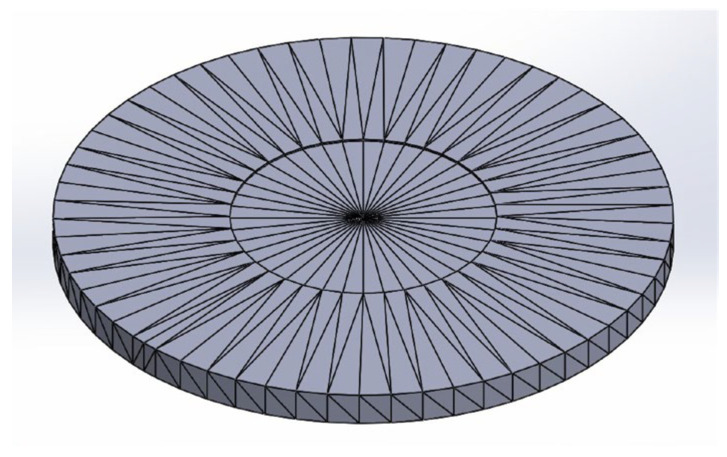
CAD of the sample.

**Figure 2:**
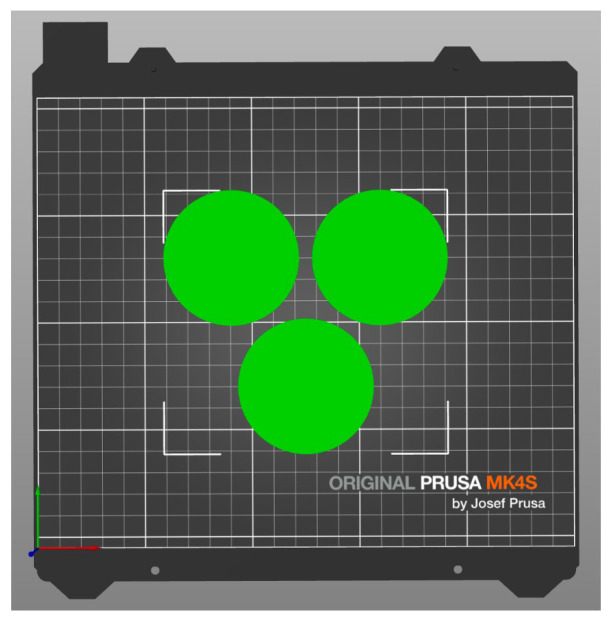
Schematics in PrusaSlicer.

**Figure 3:**
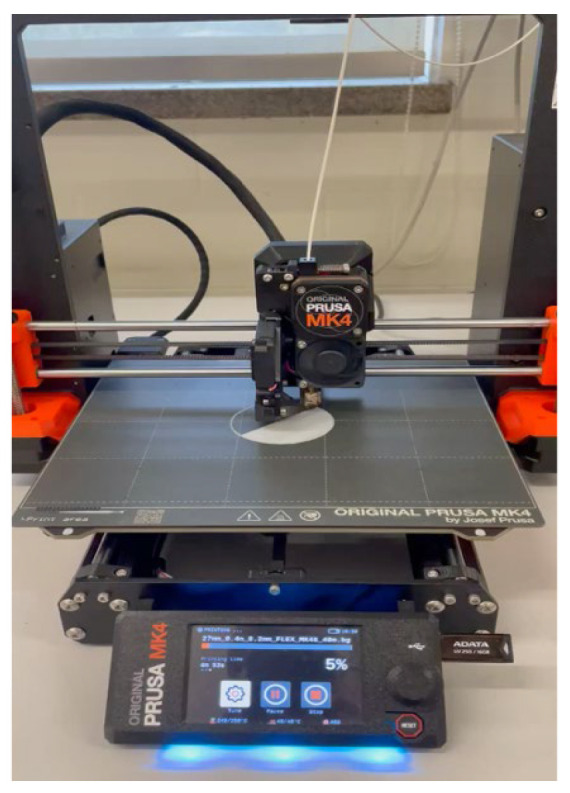
Sample printing process.

**Figure 4:**
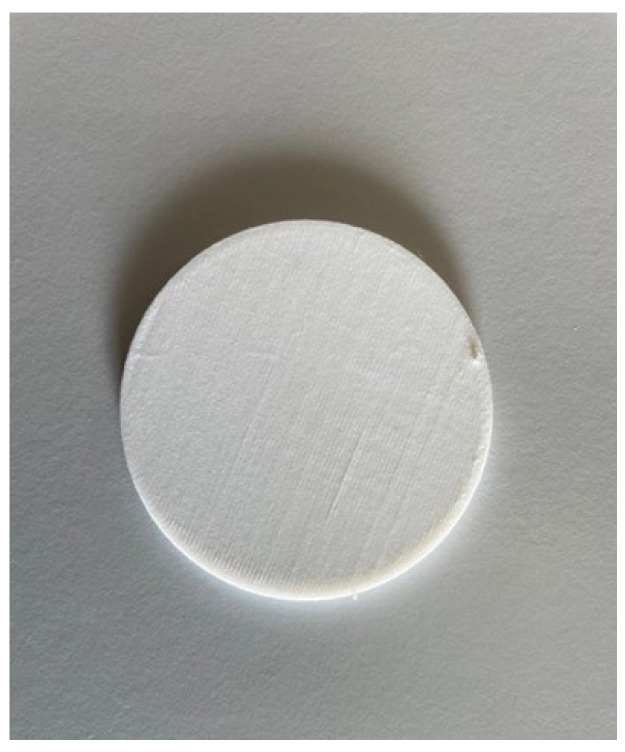
Sample without fitting.

**Figure 5:**
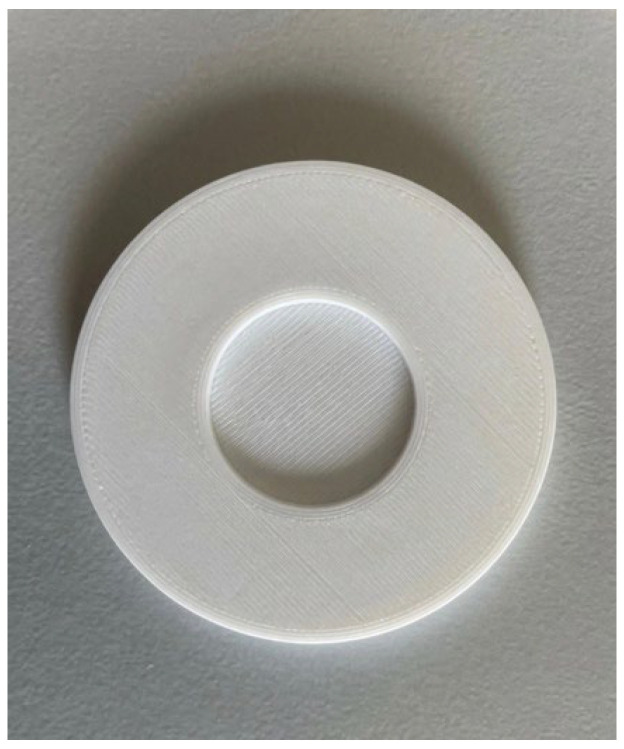
Sample with fitting.

The specimens were designed in SolidWorks 2023 (Figure 1) and then exported to PrusaSlicer v2.9.4 (Figure 2). The entire process, including printing, was conducted using a Prusa MK4 printer (Prusa Research, Prague, Czech Republic) (Figure 3). Fused Filament Fabrication (FFF) technology was applied to produce samples of TPU Filaflex Foamy 95A in a circular-prism format, both with and without fittings (Figure 4 and Figure 5, respectively).

The compression test was performed on the Shimadzu AG-10 Universal Testing Machine with a 5 kN load cell, associated with TrapeziumX software Version 1.5.1. For data cleaning and analysis, MATLAB R2025b (25.2.0.2998904) and OriginPro 2024 (10.1.0.178) were used.

As mentioned previously, to print the specimens, several parameters in the PrusaSlicer software were set. Table 1 shows some of them. The last two highlighted parameters were chosen to study the material’s behavior. For that, we set the maximum infill percentage, which required selecting the rectangular fill pattern.

The printed samples were 62.80 mm in diameter and 3.50 mm thick and were obtained on Filaflex Foamy 95 Shore A TPU. For this procedure, an Original Prusa MK4S printer with a 0.4 mm nozzle was used. The choice of this polymer was based on previous studies that indicate it is the most suitable for the intended application (development of smart insoles) and on the fact that it enables us to achieve more promising results that are in line with our final purpose. These results have already been published and relate to the behavior/results of the compression test of samples printed using FFF technology of polymeric materials PLA (poly (lactic acid)) and TPU Filaflex Foamy 95A. In the study in question, there was a rapid increase in stress and a more limited deformation capacity on the part of PLA, while TPU showed a higher energy absorption capacity [17].

Regarding the choice of the type of sensor, it was based on its ability to detect changes in force and to accommodate other actions, tasks, and movements characteristic of sports practice. For this purpose, cyclic tests were conducted to exploit the sensor’s characteristics and obtain more meaningful results for future work. This was not the case in the study of Sêco et al. [18] because the results of traditional compression tests showed atypical behavior, they failed to detect the gradual force exerted during the test. Table 2 summarizes the key specifications of the piezoelectric sensor selected for this investigation.

**Figure 6:**
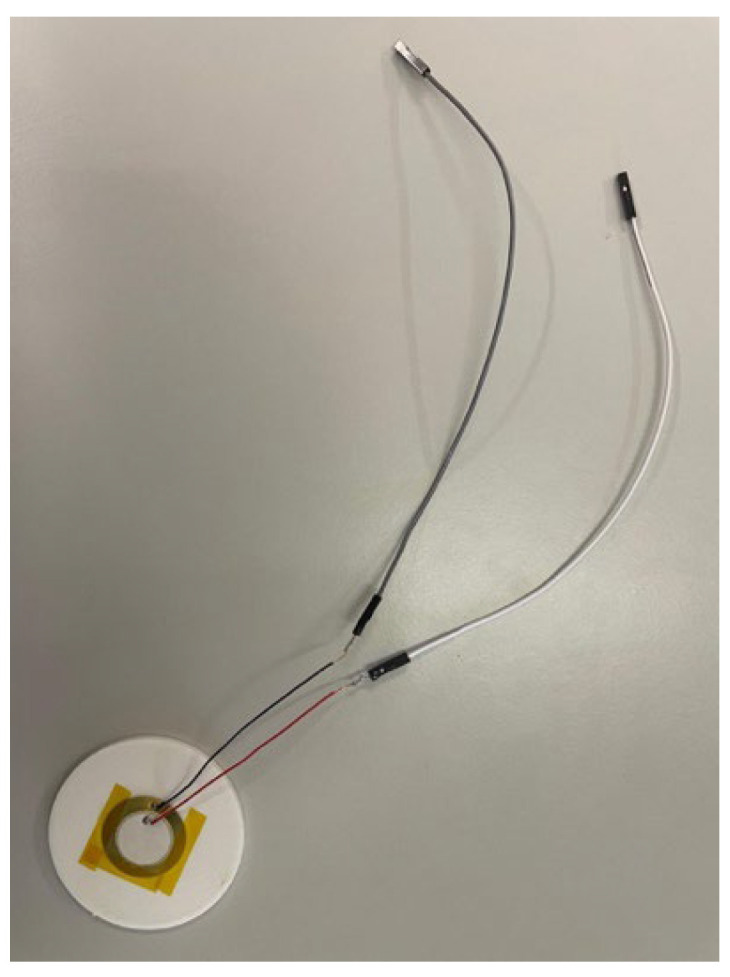
Prototype without cavity.

**Figure 7:**
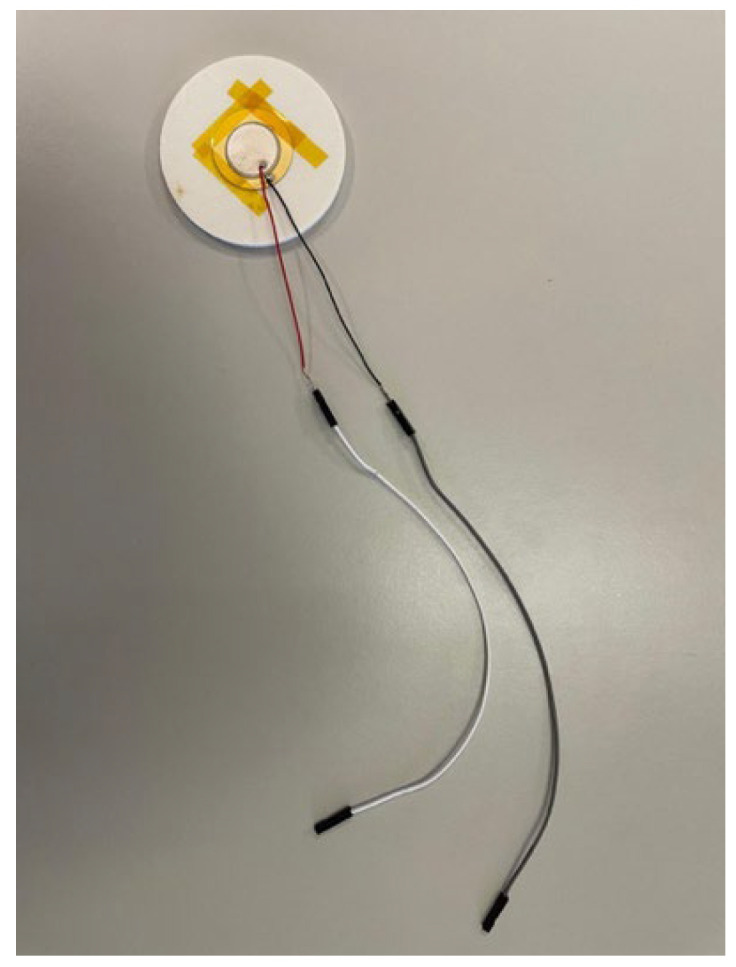
Prototype with cavity.

For this research, two prototypes were built, one prototype without a cavity (Figure 6), where the sensor was placed in the center of the sample and fixed with Kapton tape, and another prototype with a cavity (Figure 7) with dimensions of 0.2 mm depth and 27 mm diameter, in which the sensor was placed in the respective space and also fixed with Kapton tape. It is known that adhesives can reduce sensitivity by increasing stiffness and reducing strain transfer; in summary, different encapsulation designs may affect the low and high-frequency responses differently [19].

Knowing that embedded sensing in composites requires special attention to wiring, edge trimming, and system-level integration issues [20], it was decided to not overcomplicate the mounting and construction of the prototypes.

After integrating the sensor into the sample, the wires of the sensor were connected to two Male–Female (M-F) jumper wires that were after connected to A0 and GND ports of an Arduino Nano 33 BLE Rev2 microcontroller. Next, the microcontroller was connected via a USB-micro-USB cable to a 2022 Apple M2 MacBook Pro 13′ computer.

**Figure 8:**
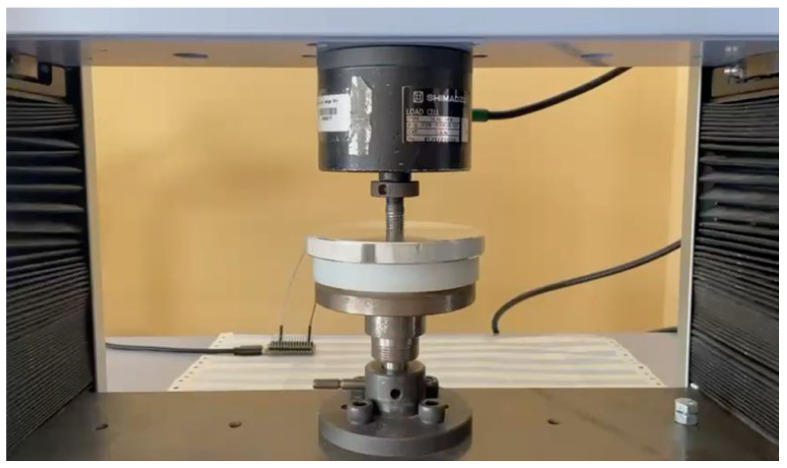
Image of the compression test process.

Each test included ten loading and unloading cycles to 1000 N, at a speed of 0.5 mm/s. The maximum displacement was held constant for 1 s at each load until the load cell reached 1000 N. Data from the sensors were collected using Python v1.109.0 code and processed in Visual Studio Code v.1.107.0. In Figure 8, it is possible to observe an image of the compression test process.

## 3. Results and Discussion

In this study, material data, including stress and strain, were collected using software. Sensor data were also gathered using Python code in Visual Studio Code, including ADC (analogue-to-digital converter), v_raw (gross tension), and time, as usual.

**Figure 9:**
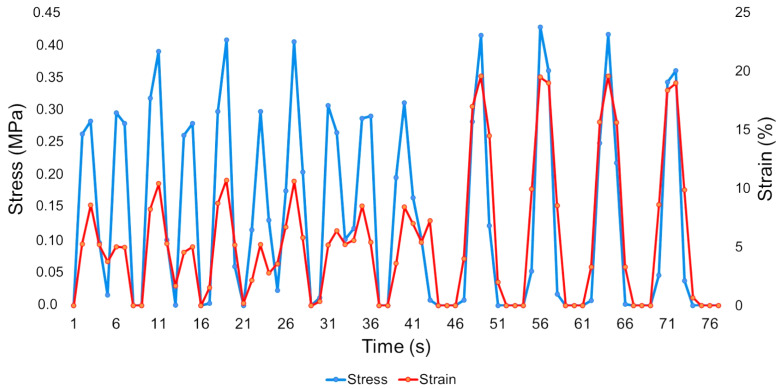
Average of the stress–strain results of the sample without a cavity.

**Figure 10:**
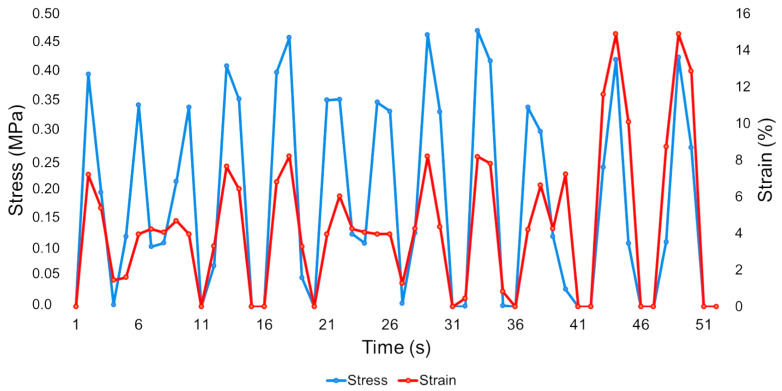
Average of the stress–strain results of the sample with a cavity.

Figure 9 and Figure 10 show the behavior of the prototype materials without and with a cavity, respectively, with stress (left *y*-axis) and strain (right *y*-axis) plotted over time (*x*-axis). Figure 9 demonstrates that a greater volume of data is present and concentrated from the beginning of the trials up to 41 s. Beyond this point, fewer data points are observed, and the remaining points exhibit similar patterns, indicating a strong correlation between stress and strain. The reduction in data points after the 41-s mark also corresponds to increased stress and strain values during the final cycles of the tests.

On the other hand, Figure 10 visually shows instantly that the points are more evenly distributed over time, so without the concentration of data points, the cycles become easier to identify. The stress data is higher than the strain throughout most of the test, but in the last two cycles, when the correlation between the two metrics is higher, the strain exceeds the stress.

Comparing the two images, some conclusions can be drawn. In Figure 9, all tests take 76 s to identify the 10 charge–discharge cycles, and in Figure 10, it is evident that the prototype can complete the identification in only 51 s, which is 25 s less than the others. The correlation between the two metrics is clearly visible in both images. In the prototype without a cavity, it increases in the last four cycles, and in the prototype with a cavity, it shows a larger correlation in the last two. Finally, with the same number of cycles and the same force applied, both prototypes achieved different results. The prototype without a cavity, across the seven tests, could exceed 0.4 MPa and approach 25% strain, while the prototype with a cavity achieved stress levels of more than 0.45 MPa and approximately 15% strain in all compression tests.

**Figure 11:**
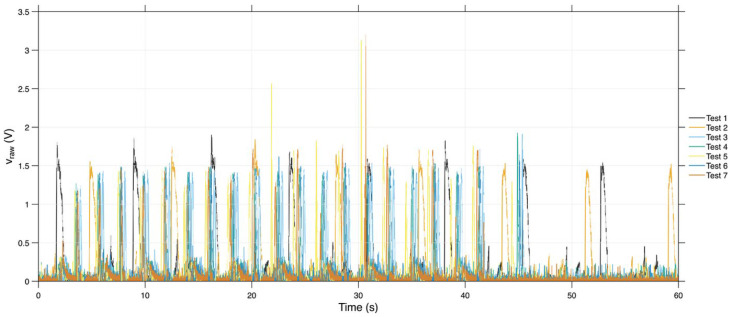
Gross tension (v_raw) in the seven tests of the prototype without a cavity.

Regarding the gross tension or voltage of the sensors, the prototype without fitting showed similar signal peaks across the charge–discharge cycles in all tests, with the signal predominantly between 1 V and 2 V. In Figure 11, where the signals from the piezoelectric sensor are represented in the seven tests with the sample without a fitting, it is possible to verify the peaks corresponding to the applied loads. However, within the 60 s stipulated time, the respective sensor recorded only 8 charging and discharging cycles in tests 1 and 2. It is also clear that test 1 showed higher peaks across all tests, reaching its peaks between 1.5 V and 2 V. The sensor in tests 3, 4, 5, 6, and 7 required less than 50 s to identify/record the ten cycles.

**Figure 12:**
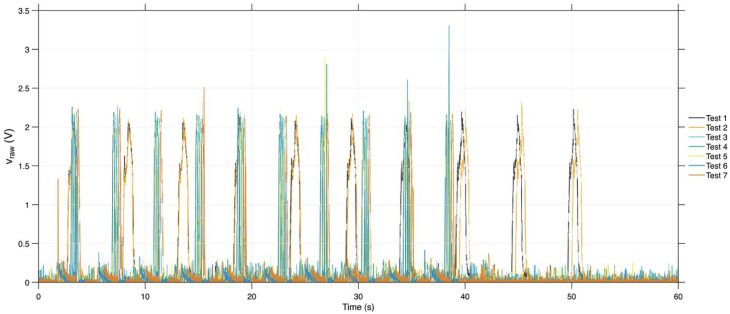
Gross tension (v_raw) in the seven tests of the prototype with a cavity.

During the piezoelectric sensor tests of the sample with a cavity, as illustrated in Figure 12, the sensor recorded ten charge and discharge cycles within 60 s, consistent with previous findings. The maximum values in all tests were predominantly between 2 V and 2.5 V. Only a few instances exceeded 2.5 V, with a single occurrence above 3 V.

**Figure 13:**
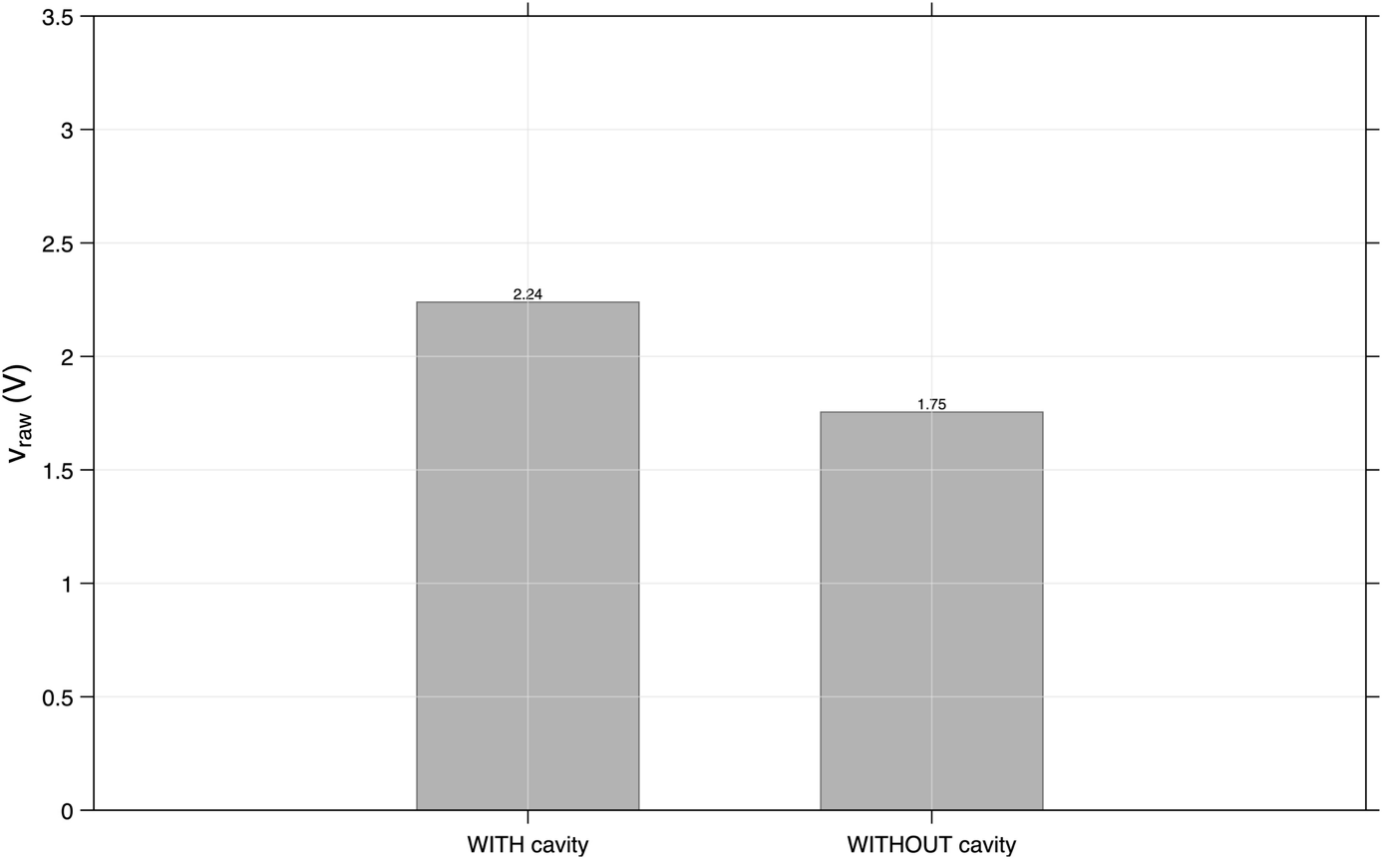
Average gross tension (v_raw) of the higher peaks.

About more global or general metrics, in Figure 13, it is evident that, when accounting for the average gross voltage (v_raw) of the 70 peaks (or maximum values), the prototype with fitting or cavity achieved 2.24 V, and the prototype without fitting achieved 1.75 V. The study by Feng et al. [21] showed that surface-mounted signals had higher amplitudes than embedded signals in many cases, the opposite of our findings. However, the cavity condition showed improvements in stability/noise [16,21].

**Figure 14:**
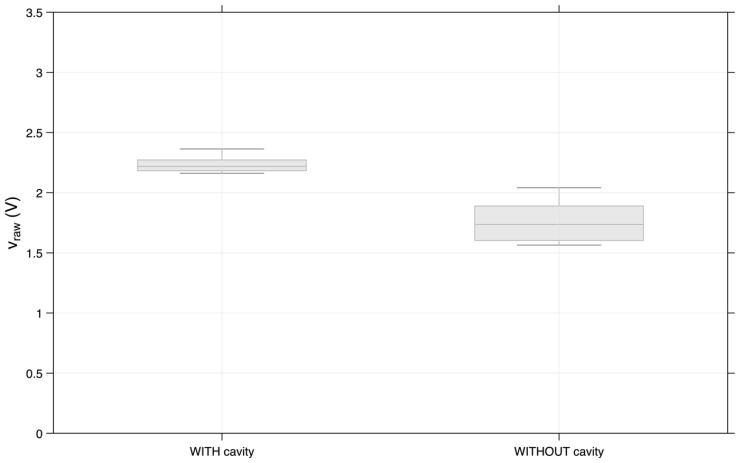
Standard deviation of the higher peaks (v_raw).

Figure 14 shows the standard deviation of the sensor’s signals for the higher peaks in all trials. The data presented next confirms what was previously stated: the prototype with a cavity can detect higher voltage peaks, ranging from 2 V to 2.5 V, and most of the peaks are very concentrated in the interval approximately 2.2 V to 2.3 V. The prototype without a cavity presented lower v_raw limits and a higher standard deviation compared with the prototype with a cavity.

**Figure 15:**
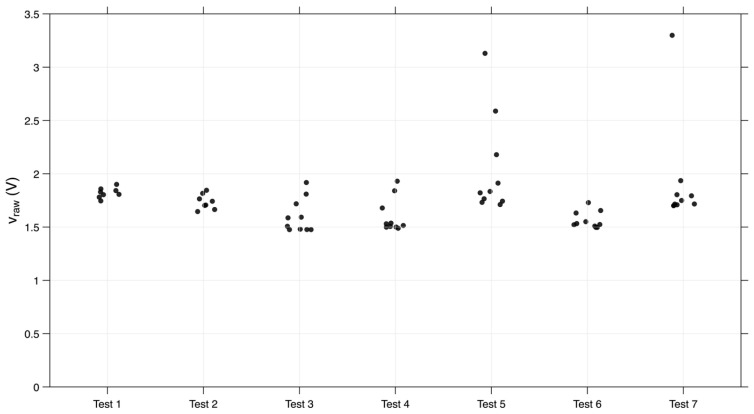
Distribution of the higher peaks (v_raw) in the seven trials of the prototype without a cavity.

More specifically, Figure 15 presents the distribution of the highest peaks recorded across all seven trials of the prototype without a cavity. Most peaks fall within the range of 1.5 V to 2 V. Higher values were observed only in tests 5 and 7. In test 5, one peak exceeded 2 V, another surpassed 2.5 V, and the final peak was above 3 V. In test 7, the maximum value recorded across all tests was approximately 3.3 V.

**Figure 16:**
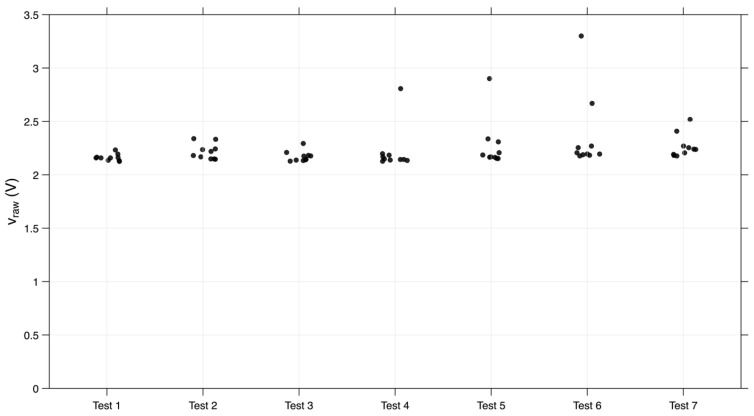
Distribution of the higher peaks (v_raw) in the seven trials of the prototype with a cavity.

In Figure 16 is possible to observe the higher-voltage peaks between 2 and 2.5 V. Among the 70 peaks recorded in seven tests, with ten peaks in each test, only five exceeded this range: four were between 2.5 and 3 V, and one peak reached approximately 3.3 V.

**Figure 17:**
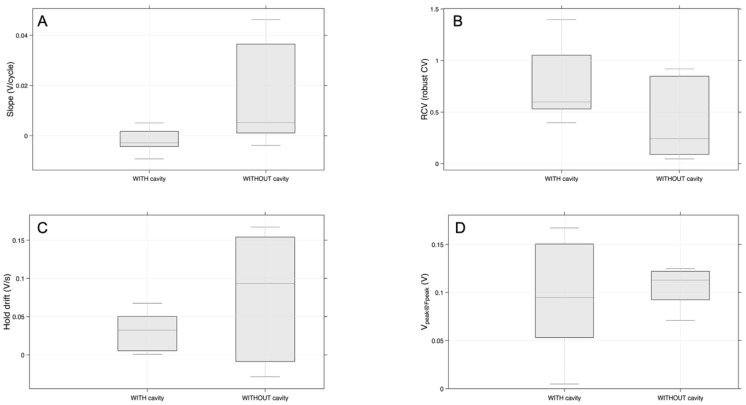
(**A**) Distribution of drift across the 10 cycles (slope) for samples with a cavity versus without a cavity. (**B**) Repeatability comparison (RCV) of hold mean |V| metric for samples with a cavity versus without a cavity. (**C**) Per-test median signal drift during 1 s. (**D**) Per-test median peak voltage at the peak-force instant, v_peak@Fpeak (V), for samples with a cavity versus without a cavity.

Based on the boxplots in Figure 17, the most striking contrast is that samples without a cavity consistently show greater signal drift and lower peak-related response, whereas samples with a cavity exhibit greater repeatability in the hold-response metric. This clearly differentiates the two groups in terms of signal stability and response variability.

Figure 17A, which shows the distribution of drift across the 10 cycles, the group without a cavity has a clearly higher median and a much broader interquartile range than the group with a cavity. This suggests that the signal slope, or cumulative drift across cycles, is generally larger in samples without a cavity. The wider spread also indicates greater variability between tests. Although the non-cavity samples tend to drift more, that effect is less tightly controlled. In contrast, the cavity group is clustered closer to zero and even includes slightly negative values. This suggests lower drift and a more stable signal trend across cycles. In Figure 17B, the situation is reversed. The RCV of the hold mean is generally higher for the samples with a cavity, while the samples without a cavity show a lower median and broader asymmetry. Interpreting RCV as a repeatability-related descriptor, this suggests that the hold-response metric is more consistently preserved in the cavity condition. The non-cavity condition produces a less uniform hold-response pattern [22].

In Figure 17C, the per-test median signal drift during 1 s is again higher for the samples without a cavity. This reinforces the pattern already seen in Figure 17A. The median is visibly larger, and the distribution extends to higher values. This indicates that the non-cavity condition is associated with stronger short-term drift. By contrast, the cavity samples remain concentrated at lower values, showing a more restrained drift response. The median peak voltage at the peak-force instant, v_peak@Fpeak, represented in Figure 17D, appears slightly higher for the group without a cavity. Here, though, the difference is smaller, and the distributions overlap more. The cavity samples appear more dispersed, while the non-cavity samples are more compact around a moderately high level.

**Figure 18:**
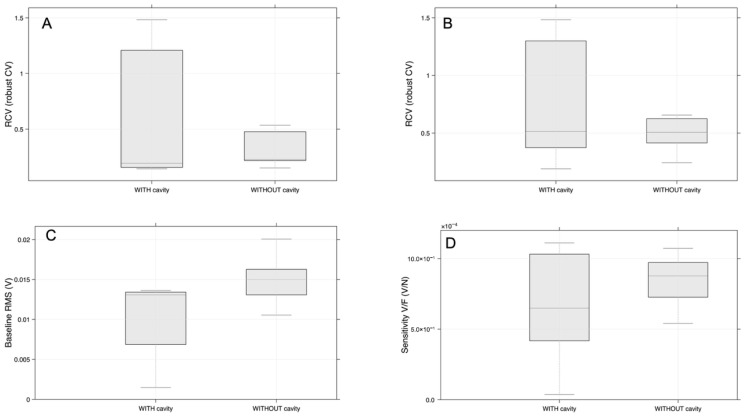
(**A**) Repeatability comparison using robust coefficient of variation (RCV) for baseline RMS: with cavity versus without cavity. (**B**) Repeatability comparison using robust coefficient of variation (RCV) for V_{peak@Fpeak}: with cavity versus without cavity. (**C**) Per-test median baseline RMS voltage (V) for samples with cavity versus without cavity. (**D**) Per-test median sensitivity (V/N) comparing measurements with cavity versus without cavity.

Figure 18 shows that the prototype with a cavity has more variation in repeatability metrics, which measure consistency across repeated tests. Without a cavity, the prototype demonstrates higher average baseline RMS (root mean square, or average baseline signal amplitude) and sensitivity (signal change when a stimulus, reaction, or force is applied), and a tighter distribution in some instances. The cavity does not affect all metrics evenly; it influences both the values and the stability of signal descriptors across repeated measurements. These results could be explained using more dynamic force parameters during the compression test.

Figure 18A demonstrates that the RCV (relative coefficient of variation) for baseline RMS is more dispersed in the cavity samples while the group without a cavity displays a more compact distribution, centered at lower RCV values. In Figure 18B, which presents the RCV for V_{peak@Fpeak} (the peak voltage measured at the frequency of the peak response), a similar but less pronounced pattern is observed. The cavity group again exhibits a broader spread, reflecting greater variability between tests, while the no-cavity group is narrower and more concentrated around intermediate values. Although this does not conclusively establish the superiority of the non-cavity sample, it does indicate greater consistency in the peak-voltage response.

Figure 18C shows that the median baseline RMS voltage per test is higher for samples without a cavity. Both the median and the upper half of the interquartile range are shifted upward compared to the cavity group. This finding indicates that the non-cavity condition is associated with a higher baseline signal level. In contrast, cavity samples exhibit a lower central tendency and extend to much lower values, suggesting that the cavity reduces the baseline RMS response and decreases its uniformity.

Figure 18D demonstrates that the per-test median sensitivity (V/N) is generally higher for the group with a cavity. This indicates that, under the dynamic loading protocol, the cavity design converts the applied force into a larger average electrical response. Combined with lower drift, the higher median sensitivity suggests that the cavity configuration is a more reliable option for detecting small force variations in wearable applications [23].

A quantitative comparison of the two sensor configurations was performed using the following experimental descriptors: sensitivity, baseline root-mean-square (RMS), hold drift, peak voltage, RCV peak, and slope cycle. Given the limited number of replicates and the non-Gaussian distribution of the data, the results were summarized as median and interquartile range and analyzed using the Mann–Whitney test, as shown in Table 3. The configuration with a cavity exhibited a lower median sensitivity compared to the configuration without a cavity, decreasing from 8.77 × 10^−5^ to 6.49 × 10^−5^ (V/N), corresponding to a reduction of approximately 26.0%. The cavity configuration also showed lower baseline RMS (0.015–0.013 V) and reduced hold drift (0.093–0.032), which suggests improved signal stability under the tested conditions. The median peak voltage decreased from 0.113 to 0.095 V, while the RCV peak remained similar between groups: 0.506 V without a cavity and 0.514 V with a cavity. Among the evaluated descriptors, baseline RMS and slope cycle provided the strongest differentiation between configurations in this dataset, with *p*-values of 0.032 and 0.029, respectively.

These results indicate that the cavity modifies the sensor response, producing a trade-off between response amplitude and stability-related metrics. In the present dataset, the cavity was associated with reduced sensitivity, lower peak voltage, fewer baseline fluctuations, and a more stable cycle trend. This behavior is qualitatively consistent with previous studies showing that cavity or backing conditions alter the effective stiffness, acoustic loading, sensitivity, and bandwidth of diaphragm-based piezoelectric sensors and transducers. For example, Liu et al. [24] analyzed the influence of an air-backed cavity on diaphragm sensitivity and bandwidth, while Dong et al. [25] showed experimentally and theoretically that cavity dimensions affect the effective stiffness and sensitivity of dynamic pressure sensors. Related PMUT studies have likewise shown that back-cavity or backing-layer modifications change output level and bandwidth, confirming that the backing condition is a relevant structural parameter in piezoelectric transduction [26].

These descriptors quantitatively highlight the effects of cavities on performance but are not intrinsic electromechanical coefficients like ^d^_33_, the coupling factor, or the mechanical quality factor. Determining those coefficients requires impedance or frequency-response characterization, which is beyond this dataset’s scope.

During cyclic compression of the prototype without a cavity, the piezoelectric output (v_raw, red trace) shows repeated high-amplitude peaks. These peaks largely coincide with the loading phases and peak stress levels (black trace), indicating that the sensor responds to mechanically induced force. However, the voltage signal also contains frequent sharp transients not mirrored by proportional changes in the average stress waveform. This suggests that the electrical response is not governed solely by the absolute stress magnitude. This behavior is consistent with piezoelectric sensing dominated by dynamic components, such as rapid stress or strain changes. It also aligns with additional contributions from unstable mechanical coupling at the sensor–polymer interface in the absence of a dedicated cavity, such as micro-drift, local strain heterogeneity, or contact intermittency.

During cyclic compression, the stress waveform (black trace) shows a sequence of well-defined load–unload cycles, while the piezoelectric output (v_raw, red trace) displays pronounced voltage peaks that occur predominantly during the stress ramp and near the stress maxima. Compared with a noisier response, the voltage trace here appears more cycle-synchronous: the largest electrical peaks tend to align with the rising portions of the stress cycles; the signal generally returns close to baseline during unloading and low-stress intervals. This pattern is consistent with piezoelectric sensing dominated by the dynamic component of loading (i.e., rapid stress/strain changes) rather than the static stress level, and it suggests a more stable mechanical coupling between the sensor and the polymer substrate during repeated cycles. Nevertheless, smaller secondary transients are still visible in several cycles, indicating that local effects, such as heterogeneous strain distribution within the polymer and minor contact/interfacial micro-motions, may still contribute to cycle-to-cycle variability. For rigorous comparison, these observations motivate reporting cycle-anchored features (e.g., peak |v_raw| in a fixed window around the stress maximum, baseline RMS, and drift during the hold portion) to quantify sensitivity, noise, and repeatability across the full set of cycles.

**Figure 19:**
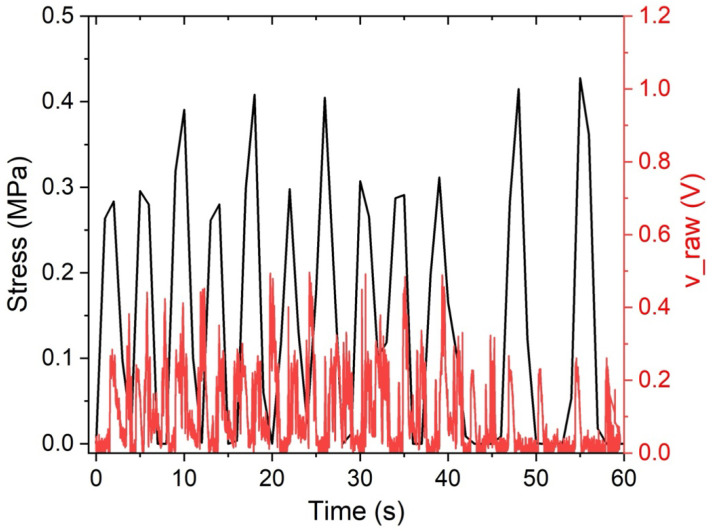
Correlation between the stress average of the prototype material vs. the gross tension (v_raw) average of the sample without a cavity.

**Figure 20:**
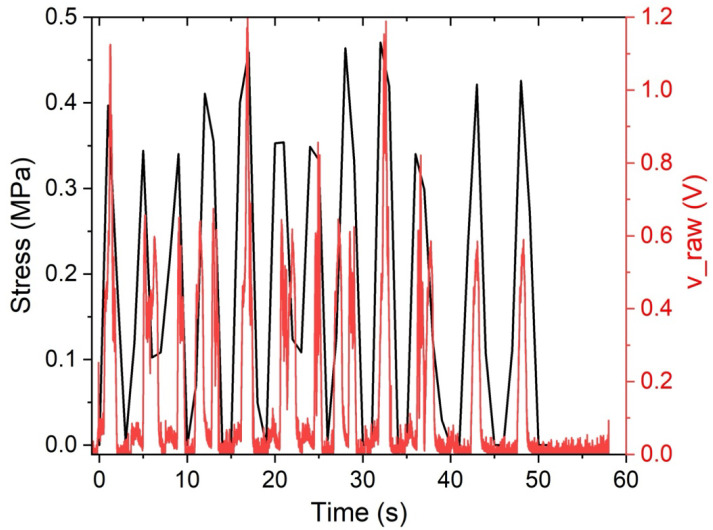
Correlation between the stress average of the prototype material vs. the gross tension (v_raw) average of the sample with a cavity.

The differences observed in the timing of the electrical response, including the apparent phase shift between the mechanical loading waveform and the PZT output, can be attributed to several mechanical and electromechanical mechanisms associated with encapsulation-dependent boundary conditions. First, the cavity and non-cavity configurations exhibit distinct local mechanical impedances and load paths, which affect both the rate and efficiency of strain transfer to the ceramic element [6]. In the cavity configuration, the sensor is laterally constrained by the cavity walls and locally supported by the surrounding TPU, reducing interfacial slip and enabling a more direct transmission of rapid stress and strain gradients. In contrast, the surface-mounted configuration depends on a tape or adhesive layer and the top TPU, introducing damping, shear lag, bending, occasional partial lift-off, and micro-slip. Adhesive and bonding layers are known to act as shear transfer media that significantly influence electromechanical coupling, amplitude, and phase response due to their compliance and thickness [27,28,29]. Moreover, the inherently viscoelastic behavior of the polymeric encapsulation materials introduces time-dependent stress–strain transfer, including stress relaxation and creep phenomena, which delay strain transmission and dissipate mechanical energy, thereby contributing directly to phase lag and temporal broadening of the electrical response [30]. These effects act as mechanical filters, attenuating high-frequency components, broadening the temporal response, and delaying the voltage peaks, consistent with studies showing that added damping layers reduce vibration-induced signal sharpness and increase noise filtering [12,31]. Second, the cavity serves as a geometric discontinuity that reflects and scatters stress waves, thereby altering the local stress field at the sensor and contributing to sharper, more synchronous electrical peaks in the embedded configuration. Dynamic models of embedded PZT transducers demonstrate that surrounding material properties and encapsulation layers significantly modify stress transfer, amplitude, and phase characteristics of the signal [32]. Third, because piezoelectric charge generation depends strongly on the rate of strain change, the preservation or filtering of high-frequency strain content produces distinct dynamic electromechanical coupling behaviors in each configuration. Experimental studies confirm that piezoelectric output is directly related to strain variation and is frequency-dependent, with higher frequencies altering sensitivity and reducing hysteresis effects [33]. Furthermore, sensor performance varies across frequency ranges depending on structural integration and boundary conditions, reinforcing the role of encapsulation in shaping signal timing and amplitude [34]. Together, these factors provide a physically grounded explanation for the encapsulation-dependent phase shift and the timing differences observed in Figure 19 and Figure 20.

Building on the mechanisms discussed above and complementing the qualitative observations in Figure 19 and Figure 20, Table 2 provides a quantitative comparison of the two encapsulation strategies. Analyzing the Table 2 values reveals that the prototypes with a cavity and without a cavity display two distinct signal profiles, as indicated by several key metrics. The most effective metrics for differentiating these classes are Hold Mean Median, Baseline Root Mean Square Median, Slope Cycle, Robust Coefficient of Variance Hold Mean, and Robust Coefficient Variance Volt Peak. Specifically, Hold Mean Med, which represents the signal level during the hold phase and is likely the most stable portion of the cycle, remains more elevated in the absence of a cavity. Baseline rms med is also generally higher without a cavity, reflecting increased baseline energy or fluctuation. Additionally, slope cycle tends to be more positive or higher, indicating a stronger upward trend in the signal over the cycle. Collectively, these metrics demonstrate that samples without a cavity exhibit a more elevated and progressively increasing signal pattern.

Conversely, samples with a cavity are more effectively identified by received-response metrics, particularly Rcv hold mean and Rcv Vpeak. These metrics are frequently higher in the presence of a cavity, indicating that the received signal exhibits greater amplitude and a sustained response during the hold region. Simultaneously, cavity samples tend to display lower hold mean med, lower baseline rms med, and lower or more negative slope cycle values. This suggests that, while the signal’s hold level and baseline characteristics are reduced, the received response becomes more prominent in specific features. This pattern can be interpreted as a shift in signal behavior due to the cavity condition, with the overall waveform level decreasing while reception-related features are enhanced. This distinction demonstrates that the cavity condition is characterized not by uniformly higher or lower values but by a redistribution of signal characteristics across different measurement components. This effect is particularly significant for health and sports applications. In these settings, key targets include gait events, posture transitions, joint movement phases, and variations in impact or loading during repetitive activities. Enhanced peaks and hold-response features may offer greater utility than a higher baseline or hold level as they facilitate improved discrimination of transient mechanical events. Therefore, the cavity condition should be regarded as application-dependent rather than a universally optimal solution.

In summary, certain variables, such as signal sensitivity median and signal-to-noise in decibels median, exhibit excessive inconsistency and are not suitable as primary indicators. That finding follows the study of Tuloup et al. [10], where it could be associated with the affirmation that PZT sensors presented several constraints, like the trade-off between stability and durability when compared with polymer alternatives. The most robust interpretation is that samples without a cavity are primarily characterized by high hold mean med, high baseline rms med, and high or positive slope cycle. In contrast, samples with a cavity are best identified by high Rcv hold mean and high Rcv Vpeak, along with lower values in hold-related metrics. Practically, hold mean med serves as the most effective metric for identifying samples without a cavity, while Rcv hold mean is optimal for recognizing samples with a cavity. These two metrics, supported by slope cycle, baseline rms med, and Rcv Vpeak, offer the most reliable and technically interpretable basis for distinguishing between the two sample types in the presented dataset. Just as they were used in the Seminara et al. [35] and Qing et al. [36] investigations to discuss repeatability and stability, these robust statistics (median/RCV/baseline RMS/drift) were also chosen to handle variability. Although it is important to know that piezoelectric sensors have different coefficients at low vs. higher frequencies [13]. Plus, the stability/noise metrics could potentially explain why additional layers and adhesives reduce strain transfer and alter electromechanical coupling and the cavity constraint behaves like partial encapsulation, allowing reduced sensitivity but improved stability, resulting in a lower baseline noise and lower drift [19].

Resuming, polymer-based piezoelectric systems, their processing methods, and mechanical state influence the effective electromechanical response. This observation aligns with the documented differences between surface and cavity mounting strategies [11]. It is also evident that the microstructure of the samples and their respective processing routes can change the output and noise. Because the mechanical boundary conditions were different, the signal quality probably changed as well [37]. Another investigation reports that built-in PZT sensors can yield simpler wave modes, implying that boundary conditions alter measured responses [15]. It also affirms that a cavity can modify constraints and alter amplitude, baseline noise, and repeatability, what was observed in this study [15]. Signal amplitude and noise can be strongly affected by transducer choice like we mentioned before. For that reason, it is obvious that this choice influences not only peak amplitude but also noise/stability metrics (baseline RMS, hold drift, and RCV) [38].

This work is essentially a controlled comparison of integration strategies to understand how mounting (without cavity vs. with cavity) can change variability and reliability [39] as it can be observed in Table 4; this is exactly the kind of practical issue this review covered by this review, specifically these problems and subject that the industry needs to address, just like the implementation issues and potential solutions. So, the path chosen for 3D printing plays a critical role in integrating functional materials into biomedical and sports sensing systems, and the integration process presents several challenges and opportunities [4].

## 4. Conclusions

The configuration incorporating a cavity reached its peak within a shorter time interval, demonstrating a faster response by detecting all ten predefined charge–discharge cycles in under 60 s.

In contrast, the configuration without a cavity exhibited a broader distribution of raw signal values (v_raw) over time. Within the same 60-s interval, this prototype detected only eight charge–discharge cycles during the initial two tests. The configuration without a cavity shows higher drift-related metrics and a slightly higher peak-voltage response, whereas the configuration with a cavity shows greater repeatability in the hold mean response and generally lower drift.

The most robust technical interpretation is that the absence of a cavity is associated with a higher baseline root mean square (RMS) value and increased sensitivity, primarily because the sensor is positioned above the sample and on the surface, resulting in greater exposure during testing. Conversely, the presence of a cavity leads to greater signal dispersion, which indicates reduced repeatability and lower central values in amplitude-related descriptors. This could be explained by the sensitivity to detect the presence and absence of applied forces.

This study demonstrates that the presence of a cavity in the sample and the proper integration of the sensor play a critical role in the dynamic transfer of load, impact sensitivity, and peak magnitude. The analysis of the raw signal highlights how structural design and sensor placement directly influence the accuracy and responsiveness of the sensing system. These findings provide important insights for the optimization of sensor-integrated insoles and contribute to the development of more reliable wearable sensing systems. Consequently, this work represents a significant advancement toward improved sports performance assessment, injury prevention, and health-related biomechanical monitoring.

## Figures and Tables

**Table 1:** Printing parameters.

Material Type	Printing Temperature (°C)	Layer Thickness (mm)	Print Speed (mm/s)	Fill Pattern	Fill Density (%)
TPU Filaflex Foamy 95A	250	0.2	45	Rectangular	100

**Table 2:** Sensor specifications.

Sensor Type	External Diameter (mm)	Resonant Frequency (kHz)	Resonant Impedance (Ω)	Static Capacitance	Plate Material	Storage Temperature (°C)	Operating Temperature (°C)
Piezoelectric Sensor (PZT)	27 mm ± 0.1 mm	4.1 ± 0.5 kHz	≤300 Ω max	28,000 pF ± 30%	Brass	−30 °C to +70 °C	−20 °C to +70 °C

**Table 3:** Quantitative metrics comparison between the two samples.

Metric	With Cavity, Median [IQR]	Without Cavity, Median [IQR]	Relative Change (%)	*p*-Value	Cliff’s δ
Sensitivity, S (V/N)	6.49 × 10^−5^ [4.86 × 10^−5^, 9.67 × 10^−5^]	8.77 × 10^−5^ [7.30 × 10^−5^, 9.68 × 10^−5^]	−26.0	0.535	−0.224
Baseline RMS (V)	0.013 [0.008, 0.013]	0.015 [0.0135, 0.016]	−13.3	0.032	−0.694
Hold drift	0.032 [0.0125, 0.044]	0.093 [−0.0095, 0.153]	−65.6	0.701	−0.143
V_{peak@Fpeak} (V)	0.095 [0.0645, 0.1415]	0.113 [0.0945, 0.1205]	−15.9	0.805	−0.102
RCV peak (V)	0.514 [0.415, 1.116]	0.506 [0.4245, 0.5915]	1.6	0.701	0.143
Slope cycle (V/cycle)	−0.003 [−0.0045, 0.0000]	0.007 [0.0035, 0.0345]	Not meaningful due to sign change	0.029	−0.714

**Table 4:** Overall test and trial metrics.

Group	Test	N Cycles	N Cycles Valid	Vpeak Fpeak Med	S Sens Med	Baseline Rms Med	Hold Mean Med	Hold Drift Med	Snr db Med	Rcv Vpeak	Rcv Hold Mean	Rcv Baseline Rms	Slope Cycle
With cavity	1	10	10	0.042	3.489 × 10^−5^	0.001	0.004	0.005	3.901	1.483	1.396	1.483	0.005
Without cavity			8	0.071	5.408 × 10^−5^	0.011	0.006	−0.008	−0.127	0.404	0.045	0.151	0.007
With cavity	2		10	0.005	3.696 × 10^−6^	0.005	0.010	0.006	−10.612	1.483	1.126	1.483	0.003
Without cavity			8	0.090	7.236 × 10^−5^	0.013	0.051	−0.029	−4.852	0.953	0.669	1.483	−0.004
With cavity	3		10	0.087	6.235 × 10^−5^	0.011	0.016	0.032	−1.030	0.331	0.599	0.194	−0.003
Without cavity				0.124	1.074 × 10^−4^	0.020	0.151	0.155	1.080	0.526	0.919	0.536	0.005
With cavity	4			0.160	1.097 × 10^−4^	0.014	0.022	0.032	0.661	0.749	0.397	0.182	−0.005
Without cavity				0.113	9.582 × 10^−5^	0.016	0.239	−0.011	−0.959	0.657	0.068	0.218	0.039
With cavity	5			0.167	1.112 × 10^−4^	0.013	0.044	0.056	0.863	0.514	0.586	0.148	−0.004
Without cavity				0.117	8.772 × 10^−5^	0.016	0.177	0.093	0.152	0.506	0.154	0.220	0.046
With cavity	6			0.123	8.367 × 10^−5^	0.013	0.047	0.067	0.911	0.499	0.514	0.382	−0.003
Without cavity				0.125	9.776 × 10^−5^	0.014	0.245	0.151	0.400	0.445	0.243	0.228	0.030
With cavity	7			0.095	6.490 × 10^−5^	0.013	0.017	0.019	−0.462	0.189	0.825	0.143	−0.009
Without cavity				0.099	7.354 × 10^−5^	0.015	0.115	0.167	0.482	0.242	0.906	0.300	0.002

## Data Availability

The data presented in this study are available from the corresponding author upon reasonable request, as public disclosure is currently limited in view of potential future intellectual property protection.
